# Temporal dynamics of cerebellar and motor cortex physiological processes during motor skill learning

**DOI:** 10.1038/srep40715

**Published:** 2017-01-16

**Authors:** D. Spampinato, P. Celnik

**Affiliations:** 1 Department of Biomedical Engineering, Johns Hopkins School of Medicine, 720 Rutland Avenue Baltimore, MD 21205, USA; 2 Department of Physical Medicine and Rehabilitation, Johns Hopkins School of Medicine, 600 North Wolfe Street Baltimore, MD 21287, USA; 3 Department of Neuroscience, Johns Hopkins School of Medicine, 725 North Wolfe Street Baltimore, MD 21205, USA; 4 Department of Neurology, Johns Hopkins School of Medicine, 600 North Wolfe Street Baltimore, MD 21287, USA.

## Abstract

Learning motor tasks involves distinct physiological processes in the cerebellum (CB) and primary motor cortex (M1). Previous studies have shown that motor learning results in at least two important neurophysiological changes: modulation of cerebellar output mediated in-part by long-term depression of parallel fiber-Purkinje cell synapse and induction of long-term plasticity (LTP) in M1, leading to transient occlusion of additional LTP-like plasticity. However, little is known about the temporal dynamics of these two physiological mechanisms during motor skill learning. Here we use non-invasive brain stimulation to explore CB and M1 mechanisms during early and late motor skill learning in humans. We predicted that early skill acquisition would be proportional to cerebellar excitability (CBI) changes, whereas later stages of learning will result in M1 LTP-like plasticity modifications. We found that early, and not late into skill training, CBI changed. Whereas, occlusion of LTP-like plasticity over M1 occurred only during late, but not early training. These findings indicate a distinct temporal dissociation in the physiological role of the CB and M1 when learning a novel skill. Understanding the role and temporal dynamics of different brain regions during motor learning is critical to device optimal interventions to augment learning.

The ability to acquire and retain motor skills is critical to the animal kingdom. Here, we refer to motor skills as the ability to improve movement speed and accuracy with repeated practice. For instance, a novice tennis player first serves tennis balls at lower speeds and with limited accuracy, but with training is able to hit high-speed balls with improved accuracy. This skill improvement likely involves multiple stages, including an early component where rapid within-session improvements are observed and are likely a result of a rapid acquisition of task dynamics (i.e. tennis racket weight, ball conditions, etc.), and a second slower phase where memories are stored and readily available for retrieval^1^,^2^.

Several studies including functional MRI, non-invasive brain stimulation studies (NIBS) and behavioral investigations in patients with cerebellar diseases have indicated a critical role of the cerebellum (CB) earlier on during motor learning^3^,^4^,^5^,^6^,^7^,^8^,^9^,^10^. Some of these investigations also showed that after an initial increase in cerebellar activity there is a decrease of activation over time^11^,^12^,^13^,^14^. However, other studies also implicated the CB in retention or later phases of learning^15^,^16^,^17^,^18^,^19^,^20^.

Activity in the primary motor cortex (M1) has also been critically implicated during motor learning. While some studies showed increased activation with motor practice^14^,^21^,^22^, others described decreasing activity with training^23^,^24^. Similarly, behavioral and NIBS research has indicated that the CB is critically involved in the acquisition of motor tasks^4^,^7^,^10^,^25^, whereas M1 is involved in the encoding of learned movements^7^,^26^,^27^,^28^,^29^, suggesting that distinct stages of skill learning weight the cerebellar and M1 roles differently. Part of the inconsistencies across investigations might result from different studies testing different motor tasks with different techniques. Therefore, the specific temporal contributions of the CB and M1 during different stages of skill learning remains incompletely understood.

The CB is thought to contribute to motor learning by predicting and accounting for systematic changes to the body or the environment, resulting in the correction of errors on a trial-by-trial basis. Animal work has shown that this form of adaptive learning is mediated, in part, by long-term depression of parallel fiber-Purkinje cell synapse in cerebellar cortex^30^,^31^. In humans, studies using transcranial magnetic stimulation (TMS) to assess the inhibitory tone the CB exerts over M1 (cerebellar inhibition, CBI) have described changes in cerebellar excitability during motor adaptation studies^8^,^9^. On the other hand, both animal and human research have shown that motor learning elicits long-term potentiation (LTP) changes in M1, resulting in a reduced capacity to induce more LTP-like changes, a phenomenon known as occlusion^32^,^33^,^34^,^35^,^36^,^37^,^38^,^39^,^40^,^41^,^42^. Evidence for occlusion of M1 LTP-like plasticity immediately after skill learning can be assessed by applying anodal transcranial direct current stimulation (AtDCS) combined with TMS^43^,^44^. While changes in cerebellar excitability and occlusion of M1-plasticity represent physiological markers of cerebellar and M1 contributions to learning, these mechanisms have never been directly tested on the same motor skill task.

Here we sought to assess neurophysiological mechanisms in the CB and M1 of humans during early and late skill learning using TMS and AtDCS to understand the temporal contributions of cerebellar and M1 networks during skill learning. We hypothesized that CBI would change early during skill learning, whereas occlusion of LTP-like plasticity in M1 would be more prominent later on as the skill is sufficiently practiced and stored. Importantly, to understand the specificity of these markers, we also assessed other measures of cortical and intracortical excitability.

## Results

### Experimental Design

**Figure 1:**
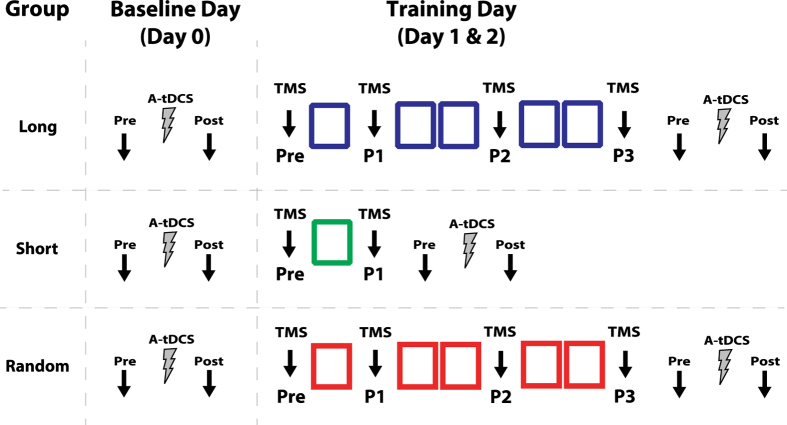
Experimental design for all groups. Long group (n = 10) individuals participated in two days of motor skill training (Day1; Day2), completing five blocks of 30 trials each day (Day1 and Day2). TMS measurements (black arrows) for this group was assessed prior to training (Pre) and after the first (P1), third (P2), and final behavioral block (P3). On each training session, individuals in the short group (n = 11) trained only on one block of the skill task and TMS physiological assessments for these individuals were recorded prior to and after completion of 1 block (Pre; P1). Random group (n = 8) was identical to the Long group, except that Random group individuals performed in a randomized version of the task. In all groups, MEP amplitudes (black arrows) were measured before and after application of A-tDCS (grey ray). This was assessed on separate days, when there was no training (Day0) and after training (Day1 and Day2).

All participants trained for two consecutive days on the sequential visual isometric pinch task (SVIPT), where squeezing a force transducer with the right thumb and index finger controls the movement of an on-screen computer cursor^10^,^27^,^43^,^44^. We randomly assigned participants to one of three distinct behavioral groups: Long (*n* = *10*), Short (*n* = *11*) or Random (*n* = *8*). On each day, the Long and Random groups completed 150 trials (5 blocks; 1 block = 30 trials) of the SVIPT, whereas the Short Training Group only completed 1 block of 30 trials (Fig. 1). In addition to motor training, all participants underwent physiological measurements to assess changes in CBI, M1 LTP-like capacity (occlusion) and corticomotor excitability (s1Mv, SICI; see the Methods below for description of each neurophysiological measurement). For the Long and Random groups, we assessed CBI, s1mV, and SICI before training was initiated, as well as after behavioral blocks 1, 3, 5 (Fig. 1, Pre-P3) on each training session (Day1, Day2). We recorded all physiological measures for the Short group only prior to and after completion of one behavioral block (i.e. early stage of learning). For each group, we measured AtDCS induced M1-LTP-like plasticity aftereffects at rest (Day0) and after completion of each training session (Day1, Day2).

### The short and long training groups learned the skill, but not the random group

**Figure 2:**
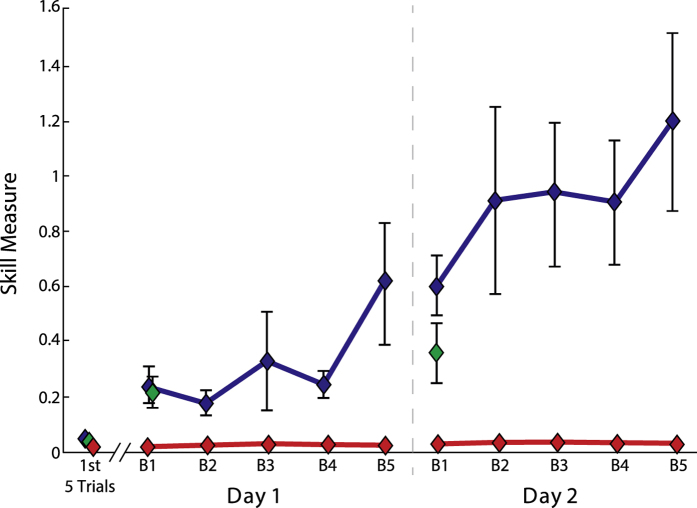
Skill learning. Skill performances are presented for the Long (blue), Short (green), and Random (red) groups. The vertical grey solid line represents the separation between Day1 and Day2 of training. The *y*-axis shows the skill measure and *x*-axis depicts the blocks of training. We also present the mean ± SEM of the first 5 trials for all groups. Note that all individuals started with similar skill level, but with more training the Long and short groups continue to improve their skill measure. Random group participants do not improve their skill measure across training sessions. Data are means ± SEM.

To assess skill learning, we compared skill measure (see equation 1 in Methods) differences across training blocks and days in the Long, Short and Random training groups. Given the different number of observations between the 3 groups, first we compared the performance of block 1 across the 3 groups for day 1 only. Performance was significantly better in the Long (*p* = *0.023*) and the Short groups (*p* = *0.020*; Fig. 2) relative to the Random Group. Moreover, there was no early performance difference between the Short and Long groups (*p* > *0.5*), suggesting that individuals from these two groups were similar at acquiring the skill. Additionally, the skill measure for all three groups was similar after the first five trials (*p* > *0.5*). This indicates that all groups started with a similar level of knowledge of the task and that learning occurs within block 1 for the Long and Short groups only. We also compared the skill measure of each block across both training sessions between the Long and Random groups, where the amount of performance was matched. ANOVA_RM_ revealed a significant difference for GROUP (*F*_(*1,16*)_ = *6.859;* p = 0.019), DAY (*F*_(*1,16*)_ = *4.878;* p = 0.042) and DAY × GROUP interaction (*F*_(*1,16*)_ = *4.779;* p = 0.044). These results show that participants exposed to a consistent sensorimotor mapping acquired the skill, whereas individuals who trained on an inconsistent mapping were unable to improve their performance.

### CBI changes are specific to early skill learning

**Figure 3:**
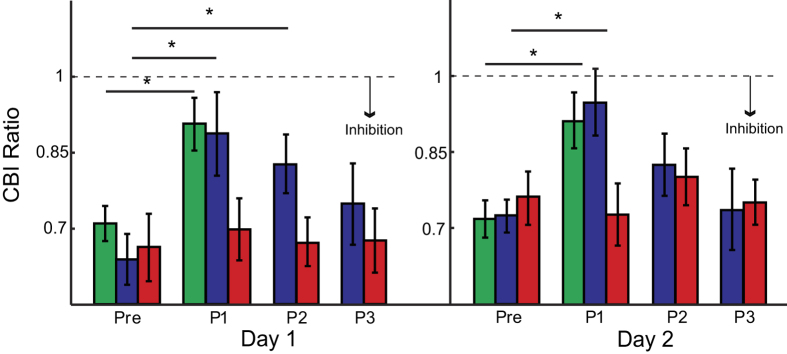
Cerebellar Excitability (CBI). Bar graphs show the mean CBI amplitude for Long (blue), Short (green), and Random (red) groups. The y-axis represents the CBI Ratio and the x-axis represents stimulation time-points before (Pre), during (P1, P2) and after (P3) training on each day. The ratio increases (less inhibition) early in learning for both the Long and Short groups, but not for the Random group. Data are means ± SEM. *p ≤ 0.05.

We compared the amount of CBI changes across the groups before, during and after the skill training blocks. Since stimulation time points before training (Pre) and after 1 block of training (P1) was matched between each group, we first assessed the early changes in CBI across block 1 for each day. ANOVA_RM_ revealed a significant effect of CBI for TIME (*F*_(*2,26*)_ = *21.106; p* < *0.001*) and TIME x GROUP interaction (*F*_(*2,26*)_ = *5.684; p* = *0.009*). CBI following early skill learning in the Long and Short groups were significantly decreased compared to Pre (*p* < *0.001, p* = *0.001*, respectively; Fig. 3), whereas we found no difference for the Random group (*p* = *0.955*). This finding shows that CBI changed early on, only when subjects learn.

To determine whether CBI returns towards baseline values late in skill learning, we additionally compared CBI between Long and Random groups across days for each time point (Pre, P1, …, P3). ANOVA_RM_ revealed a significant effect of CBI for TIME (*F*_(*3,48*)_ = *3.362; p* = *0.026*), GROUP (*F*_(*116*)_ = *7.462; p* = *0.015*) and TIME × GROUP interaction (*F*_(*3,48*)_ = *3.597; p* = *0.02*). Subjects in the Long training group showed significant CBI changes from pre-training to early skill learning (Pre-P1, *p* = *0.001*; Pre-P2, *p* = *0.003*), but no differences from pre-training to late skill learning (Pre-P3, *p* = *0.307*). This result suggests that the reduction of CBI occurs early during learning with a progressive return towards baseline despite further overall skill improvements. Interestingly, we found no significant differences in DAY × TIME (*F*_(*3,48*)_ = *0.086; p* = *0.967*) or DAY × TIME × GROUP (*F*_(*3,48*)_ = *0.163; p* = *0.921*) in the Long and Random groups, indicating the CBI dynamic changes during learning were similar across training session.

Importantly, the changes in CBI were not due to simple changes in test stimulus responses from M1. MEP amplitudes generated by the unconditioned TS were not different over TIME or SESSION in all groups (Table 1; all *p* > *0.1*). This is because we controlled for potential M1 excitability changes by adjusting the stimulator intensity. Thus, our results indicate that changes in CBI, probed by CB-M1 stimulation, are present early on within a skill training session, but not late.

### CBI changes are proportional to skill acquisition

**Figure 4:**
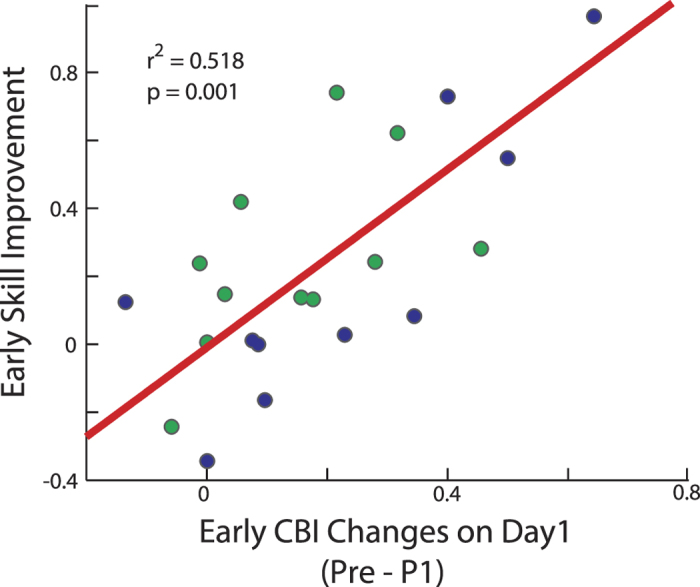
Correlation between early CBI changes and behavior. *y*-Axis represents early skill acquisition on day 1, and the *x*-axis represents early CBI change (P1-Pre). Blue circles represent individual subjects of the Long group and the green circles correspond to Short group individuals. To calculate early skill acquisition, we split the first behavioral block (B1) in half and computed two separate skill measures (first 15 trials vs. next 15 trials) and calculated the difference in skill measure. Note that both groups experienced the same amount of trials to allow for this comparison. Subjects who had the largest CBI change improved the most in the first block of D1.

Previous investigations have shown AtDCS over the cerebellum increased on-line skill learning, with the most noticeable difference after the first training block^10^. Moreover, changes in CBI were previously found to correlate with the amount of locomotor adaptation^8^. Here, we tested whether a similar relationship between change in CBI and early skill learning exists. To calculate early skill improvement, we divided the first training block in half and re-calculated two new skill measure scores for each half. Thus, we subtracted the skill score from the second half vs. the first half (within block 1 skill improvement) and performed a correlation analysis with CBI changes between pre-training and after the first training block (Pre-P1) in the Long and Short training groups. We found a relationship between skill improvement within the first block and early change in CBI (*r* = *0.518; p* = *0.001*; Fig. 4), where participants who showed more CBI changes were better at acquiring the skill.

### Occlusion of LTP-like plasticity occurs later during the skill practice

**Figure 5:**
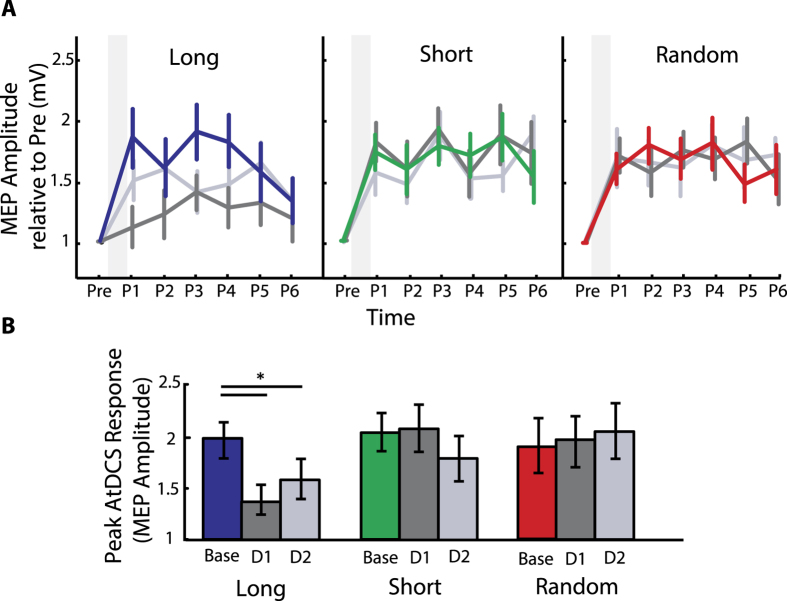
M1 LTP-Like Plasticity Aftereffects. (**A**) MEP amplitude ratios for pre and post AtDCS. y-Axis represents the mean MEP amplitude normalized to the pre-AtDCS MEP amplitude, and the x-axis presents TMS measurements taken before application of AtDCS (pre), immediately after AtDCS (Post 1, P1) and repeated every 5 minutes up to 25 min after AtDCS (P2…P6). The left, middle and right portion of the graph depicts MEP amplitudes for individuals of the Long, Short and Random group respectively. Colored lines represent the timeline of MEP amplitude responses for all subjects on Day0 (baseline session), whereas dark grey and grey present responses after Day1 and Day2 training session. The gray shadowed columns represent the time when AtDCS was applied. Note, all groups demonstrated an increase in excitability in response to AtDCS for the baseline session. (**B**) The bar graphs show the peak MEP amplitude response following AtDCS for each session. Only participants of the Long group showed significant occlusion of LTP-like plasticity after each training session when compared to baseline responses. Data are means ± SEM. *p ≤ 0.05.

We compared the amount of AtDCS-induced potentiation before and after training in the Long, Short and Random groups. We found a significant effect of AtDCS on MEP amplitudes for TIME (*F*_(*1,52*)_ = *32.892, p* < 0.01), TIME × GROUP (*F*_(*1,52*)_ = *16.618, p* < *0.01*) and DAY × TIME × GROUP interactions (*F*_(*4,52*)_ = *2.629, p* = *0.045*). All groups experienced an increase in MEP amplitudes in the baseline session (*p* < *0.01*). However, while the Short and Random groups showed similar increase in MEP amplitudes following application of AtDCS after the training (*p* < *0.01*), the Long group failed to show differences in MEP amplitudes (*p* = *0.186*, Fig. 5a). To further quantify the amount occlusion of LTP-like capacity after training, we computed an occlusion index (OI) (see equation 2 in Methods) for each group. The OI represents the difference between the peak MEP response following application of AtDCS at baseline and following AtDCS after training. We found that only the Long training group experienced occlusion (i.e. a large OI) in both days (*F*_(*1,18*)_ = *14.634, p* = *0.440*), but not the short and random training groups (*F*_(*1,20*)_ = *0.621, p* = *0.001*; *F*_(*1,14*)_ = *0.354, p* = *0.561*). Altogether, these results indicate that more skill training leads to interference or occlusion of AtDCS potentiation effects.

### Motor skill retention is proportional to the amount of occlusion

**Figure 6:**
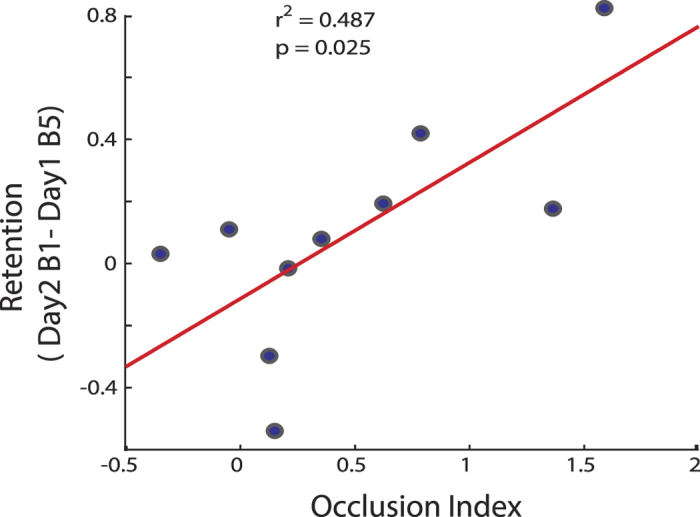
Correlation between Occlusion of LTP-like Plasticity and Behavior. Y-axis represents the retention of the skill measure and x-axis represents the Occlusion Index. Blue circles represent individuals of the Long group. Note that subjects who had the largest OI after training had the best skill retention measured on day 2.

We conducted a correlation analysis between offline behavioral changes in the Long training group (Day2 B1-Day1 B5) and the first day OI. We found that those who retained the most experienced the largest occlusion (*r* = *0.464; p* = *0.025*; Fig. 6). This finding is consistent with prior studies showing that occlusion is proportional to skill retention^43^,^44^.

### M1 excitability changes are not specific to learning

First, we compared early s1mV changes across days for block 1 in all groups. ANOVA_RM_ showed a significant effect of s1mv changes for TIME (*F*_(*2,26*)_ = *10.256; p* = *0.004*), but no differences for GROUP (*F*_(*2,26*)_ = *0.169; p* = *0.845*), DAY (*F*_(*1,26*)_ = *0.906; p* = *0.350*) or their TIME x GROUP interaction (*F*_(*2,26*)_ = *0.793; p* = *0.463*).

Second, we used ANOVA_RM_ to compare s1mV changes in the later time points in the Long and Random groups. Again, we found a significant difference for TIME (*F*_(*3,48*)_ = *6.022; p* = *0.001*), but no differences for GROUP (*F*_(*1,16*)_ = *0.229; p* = *0.639*), DAY (*F*_(*1,16*)_ = *0.122; p* = *0.732*) or their TIME x GROUP interaction (*F*_(*2,26*)_ = *0.511; p* = *0.677*). These results indicate that changes in s1mV excitability were not specific to learning, but rather due to motor execution.

Finally, we performed a similar analysis for SICI. When comparing early SICI changes for all groups, ANOVA_RM_ did reveal significant effect of TIME (*F*_(*1,26*)_ = *5.546; p* = *0.027*), but there were no differences within GROUP (*F*_(*2,26*)_ = *0.421; p* = *0.661*) or between TIME x GROUP interaction (*F*_(*2,26*)_ = *0.508; p* = *0.607*). When comparing SICI at the other time points in the Long and Random training groups, we found no significant changes (all *p* > *0.2*). Thus, our results showed that early changes in SICI are due to motor execution or the simple passage of time, but not to skill learning.

### Discussion

Our study demonstrates an important temporal dissociation in the neurophysiological role the cerebellum and M1 play during skill learning. Specifically, we found a reduction in CBI early in skill learning, but not late. The magnitude of the CBI change correlated with early skill acquisition; participants who initially improved the most on their ability to perform a new skill had the largest reduction of CBI. On the other hand, occlusion of M1 LTP-like plasticity only occurred after a significant amount of training has taken place. In addition, we found that those participants who occluded the most on their first training session had better skill retention on the following day. Critically, these changes were only observed with learning, but not when subjects performed a randomized task that does not lead to learning. Indeed, overall motor execution, rather than learning, resulted in primary motor cortex excitability changes and a reduction in SICI.

There is growing evidence from both animal and human studies demonstrating that learning induces changes in synaptic plasticity in both the cerebellum^8^,^9^,^30^,^31^,^45^,^46^,^47^ and the primary motor cortex^34^,^38^,^39^,^40^,^41^,^42^,^43^,^44^,^48^,^49^. Specifically, the cerebellum is critical to error-based learning tasks, such as motor adaptation, where motor commands are rapidly adjusted for new predictable demands^25^,^50^,^51^. On the other hand, M1 has an important role in motor retention^26^,^27^,^28^,^43^,^44^,^52^,^53^. In this region, Hebbian, such as use-dependent learning, and operant reinforcement mechanisms where learning is influenced by the repetition of prior successful movements has been described^26^,^38^,^39^,^49^,^51^. Despite the distinct roles for the cerebellum and M1 during skill learning, physiological mechanisms related to these structures have never been tested directly in the same task throughout the different stages of skill learning.

As we have previously shown in motor adaptation studies^8^,^9^,^54^, we found that CB-M1 connectivity changed early in the (long and short) skill-learning groups only, and returned to baseline levels as training proceeded. Given that we controlled for M1 excitability modifications when assessing CBI (by adjusting TMS intensities) and the fact that M1 excitability changed similarly in all groups and in all the post-training time points, we interpreted the specific CBI findings as driven by cerebellar plasticity. This suggests that error-based learning processes, which heavily contribute to motor adaptation tasks, might play a similar role early on during skill acquisition. We reason that to be able to learn a new skill first it is crucial to calibrate the appropriate motor outputs to interact with either the device being used and or the dynamics of the environment. In our experimental setup, learning the relationship between the force transducer and cursor movement could be interpreted as similar to a perturbation of a previously known map in a motor adaptation task. Participants have to account for this force-visual display map and adjust their movements to make a successful action. We therefore conclude that learning this mapping (i.e. the dynamics of the task) is what drives early engagement of the cerebellum, as denoted here by CBI changes. Indeed, we found that when participants performed the skill on an inconsistent trial-to-trial force-distance map task, CBI did not change. This finding is consistent with previous studies that found AtDCS over the cerebellum reduced the error rate when learning the same task lead to improved skill acquisition^10^, as well as visuomotor, locomotor and force field adaptation^7^,^55^,^56^. We also predicted that the early CBI changes should correlate with the amount of early skill acquisition. Of note, although we did not find an association between CBI and error rate, early CBI changes correlated with improvement in early skill measure scores, which reflects improved trade-off between error rate and movement time.

Previous animal and human investigations have shown that after learning a motor task the ability to artificially induce LTP-like changes is reduced, an effect explained by the saturation of the synaptic modification range^35^,^36^,^37^,^38^,^39^,^40^,^41^,^42^,^43^,^44^. According to this model, if skill learning uses up some of the plasticity available at motor cortical synapses, then additional synaptic strengthening through LTP-like mechanisms should be reduced. In other words, there is a reduced capacity for further LTP-plasticity after learning. We observed this phenomenon, named occlusion of LTP-like plasticity, only in participants who were able to learn the motor task. This result supports the idea that motor learning-induced plasticity interacts with NIBS protocols that lead to plastic changes in M1^38^,^39^,^40^,^43^,^44^. Consistent with Cantarero, *et al*.^43^, we also found an association between the magnitude of occlusion on day 1 and retention of the skill on the next day, supporting the evidence that occlusion is a critical mechanism of skill retention. Of note, previous studies have shown that retention of the SVIPT motor task is unaffected by AtDCS application immediately after training^43^,^44^. In addition, while recent studies have found that consecutive AtDCS sessions can lead to cumulative increases in cortical excitability^57^,^58^, we found no differences in the baseline TMS measures across days. This indicates that any potential tDCS aftereffects on excitability were not present on Day 2. Furthermore, although the intra-subject variability of AtDCS aftereffects remains poorly understood, it might be a potential limitation of the occlusion measurement. However, the lack of AtDCS potentiation was present only in the Long training group, but not in the Short and Random groups. Thus, despite this potential limitation, the effect size found in the Long group was larger and beyond the variability that can be expected in all groups. This supports the concept that occlusion of LTP-like plasticity is only observed during learning, but not with simple motor execution.

In our study, however, we found that occlusion of LTP-like mechanisms was not present early on during skill learning. A possible explanation for this result is that early skill learning may rely more on error-dependent forms of learning. This would suggest that at early stages of skill learning error-based learning is weighted more to acquire the dynamics of the task before movements are worth encoding. This is not to say that other mechanisms within M1 that support early rapid plasticity, such as unmasking of pre-existing connections^59^ or awakening of silent synapses by insertion of postsynaptic AMPA receptors^58^ are not occurring in the early stages of learning. Alternatively, it is also possible that some synaptic strengthening via LTP-like mechanisms is taking place early on during learning, but our measures to detect these changes are not sensitive enough.

In this study, we show that occlusion also occurs after significant performance improvement on the second training session. This result is in agreement with previous studies in rats, which showed occlusion of LTP plasticity up to five days after skill learning^35^. In humans, however, Rosenkranz, *et al*.^40^ failed to observe persistent LTP/LTD-like plasticity after five days of training. The authors suggested that this effect could be attributed to learning via a different mechanism other than LTP/LTD-like plasticity; however, it is also possible that in that study, little learning took place in the last session of training. This would be consistent with our random skill-training group, which did not experience learning or occlusion.

Previous studies have shown that M1 excitability and short-interval intracortical inhibition (SICI) changes with motor learning^60^,^61^,^62^. However, these investigations did not control for movement execution vs. true performance improvement. Here, we found that both the learning and non-learning groups experienced changes in corticomotor excitability and SICI. This indicates that motor execution leads to M1 excitability changes, an effect that is not necessarily tied to learning. This observation is consistent with our previous findings in healthy individuals learning motor adaptation tasks^8^,^9^. Of note, it is possible that other forms of SICI (e.g. SICI at 1 msec) not tested in this study might also modulate with learning^63^, a question that needs to be addressed in future studies. Altogether, these results indicate that occlusion of M1-AtDCS effects may be a better marker for learning-related plasticity rather than simple changes in M1 excitability or SICI.

This study demonstrates the temporal dynamic role of the cerebellum and M1 when learning new motor skills. Our results suggest that skill learning, which likely relies on many different forms of learning, incorporates early on cerebellar-dependent error-based processes, and later on engages M1-LTP like plasticity that might be linked to other forms of learning such as reinforcement or use-dependent. This is in agreement with recent work that suggests that learning different motor tasks involves error-based and other forms of learning^51^,^64^,^65^. Altogether, our findings indicate that early on during motor skill learning, cerebellar-dependent learning mechanisms (i.e. error-based process) are needed to learn the task dynamics before the primary motor cortex, incorporating other forms of learning (i.e. reward-based or use-dependent), is engaged. This concept is critical to design rational interventions that target specific neural regions to affect specific processes to augment motor learning.

## Methods

We recruited a total of 29 young, healthy right-handed individuals (mean age = 22.04 ± 0.56 years; 16 female) with no history of neurological disorders. Participants were screened prior to enrollment in the study to ensure that they did not have conditions that would exclude them from non-invasive brain stimulation. Exclusion criteria included the use of nicotine, alcohol, recreational drug use and absence of prescribed medication affecting the central nervous system, all of which may alter plasticity and motor learning. All participants provided written informed consent to participate in this study and the Johns Hopkins School of Medicine Institutional Review Board (IRB) approved all experimental procedures. All experiments were performed in accordance with relevant guidelines and regulations.

### Behavioral Measurements

#### Motor Skill task: Sequential Visual Isometric Pinch Task (SVIPT)

As previously described^10^,^27^,^43^,^44^, participants were seated in front of a computer screen and held a force transducer between the right thumb and index finger. Pinching the force transducer controlled the movement of an on-screen cursor with an overall goal to move the cursor between a HOME position and 5 targets (the sequence of movements: HOME-1-HOME-2-HOME-3-HOME-4-HOME-5). This sequence was held consistent throughout training. Long and Short training groups were exposed to a consistent and learnable logarithmic sensorimotor mapping between forces applied to the transducer and cursor movement, whereas Random group individuals were exposed to a variation of the SVIPT where the force-distance mapping and sensitivity was randomized trial-by-trial. All participants were informed that both movement time and accuracy contributed to the overall skill score and were encouraged to improve in both domains. Movement time was considered as the total time from movement onset until target 5 was reached. A trial was considered correct only if participants hit each target in the correct order. Thus, accuracy for a trial was calculated in a binary fashion (i.e. no error or error), regardless of participants committing multiple errors.

To assess the skill performance of participants, we used a speed-accuracy trade-off function (SAF)^27^. The function used to estimate the SAF throughout performance is the skill measure described in equation 1:


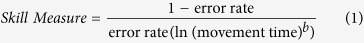


As done in previous studies^10^,^43^,^44^,^66^, average movement time and error rate (proportion of trials with at least 1 error) were calculated for each block of 30 trials, and the value of *b* was held constant at 5.424.

#### EMG Recording

Electromyographic (EMG) activity was captured using electrodes placed over the right first dorsal interosseous muscle (FDI) muscle. EMG signals were sampled at 2 kHz, amplified at 1 kHz and band-pass filtered (10–500 Hz) using an amplifier (Octopus AMT 8; Bortec Biomedical, Alberta, Canada) and data acquisition software (Signal 4.02; CED, Cambridge, England). Data was stored on another computer to complete off-line analysis using a variety of custom Matlab scripts (MathWorks, MA, USA).

#### Transcranial magnetic stimulation (TMS)

For all TMS measures, we used a 70 mm-diameter figure-of-eight TMS coil (Magstim 200^2^) over M1. We used a neuronavigation system (BrainSight; Rogue Research) to ensure stimulation over the desired M1 location occurred at the same spot from session to session. To do this, we identified and marked the spot over the M1 with the best representation of the right FDI muscle. In this location, we found the resting motor-threshold (rMT) for the FDI, or the minimum intensity needed to elicit an MEP of 50 uV on 5 out of 10 pulses^67^. The rMT values collected from individuals within each group can be found in Supplementary Table S1. For all analysis, we recorded peak-to-peak amplitudes of motor evoked potentials (MEP) using electromyography (EMG).

#### Transcranial Direct Current Stimulation (tDCS)

Using a Chattanooga Ionto Phoresor II Auto device (model PM850; IOMED, UT, USA), we delivered anodal transcranial direct current stimulation (AtDCS) through two sponge 25 cm^2^ electrodes soaked in a saline solution. Electrodes were placed on the contralateral (left) M1 corticomotor representation of the right FDI muscle and the ipsilateral supra-orbital area. Stimulation was applied for 7 minutes at an intensity of 1 mA as participants were instructed to relax and remain seated. We used AtDCS as our LTP-like inducing protocol since this form of stimulation can increase cortical excitability via NMDA receptor, BDNF and calcium-dependent mechanisms in humans^68^,^69^,^70^, and is capable of assessing occlusion after motor learning^43^,^44^.

Recent studies have indicated that AtDCS aftereffects can show variability between-subjects^71^,^72^,^73^, where some individuals do not show potentiation of MEPs after AtDCS application. We therefore screened out non-responders to AtDCS based on post-AtDCS MEP changes in the baseline session. A non-responder was classified when the mean of normalized MEP amplitude across each post-AtDCS time points did not exceed 1.1 (i.e., smaller or equal than pre-AtDCS MEP). Six individuals did not meet this criterion and were therefore excluded from the study. Importantly, the intra-subject consistency of AtDCS responses across multiple sessions is poorly understood, but might be a factor that can influence the group occlusion magnitude^71^,^72^,^74^.

#### Measures of Cerebellar Excitability (CBI)

For cerebellar stimulation, we placed a double-cone coil (110 mm mean diameter) 3 cm lateral to the inion, with the stimulator current directed downward^75^. As previously described, we assessed CBI by using a paired-pulse stimulation paradigm^8^,^9^,^54^,^75^,^76^,^77^,^78^. For each measurement, we collected 20 TMS test stimuli (TS) over left M1. In half of these exposures, selected randomly, a TMS conditioned stimulus (CS) was delivered over the right cerebellum 5 ms prior to the TS. Accordingly, a total of 10 CS + TS and 10 TS pulses were administered. CBI was calculated as the ratio of the mean MEP amplitude in the CS + TS relative to TS. The CS maximum stimulator output (MSO) for all participants was set to 70% since no MEPs were evoked via brainstem pathways at 75% MSO. Throughout each stimulation time point, the intensity of the TS was adjusted to evoke an MEP of ~1 Mv. The mean stimulus intensity for the adjusted TS throughout this study was less than 1% (see Supplementary Table S2). Thus, the CBI response reflects cerebellar inhibition of M1 regardless of changes in excitability of M1 after training.

#### Measures of Primary Motor Cortex (M1) Excitability

To assess corticomotor excitability, we determined the MSO intensity needed to evoke an MEP amplitude of ~1 mV at rest (s1mV). At this MSO intensity, we recorded 10 MEPs at each stimulation time point. Furthermore, to assess short-intracortical inhibition (SICI), we used an interstimulus interval of 2 ms between subthreshold CS and suprathreshold test stimulus for the paired pulse paradigm^79^. We set our subthreshold CS set at 80% of rMT and suprathreshold TS intensity set to elicit ~1 mV^80^,^81^. Similar to CBI, we established SICI as a ratio of 10 CS + TS over 10 TS MEPs. The intensity of the TS used to measure SICI was adjusted to the same intensity used for CBI.

#### Measures of LTP-like saturation (Index of Occlusion)

To assess occlusion after motor performance, we applied AtDCS as done in Cantarero, *et al*.^43^,^44^. On each experimental day, prior to applying AtDCS, we collected 10 MEPs at the intensity used for s1MV. If participants M1 excitability increased after training, we adjusted the s1MV intensity to elicit ~1 mV amplitude and used this adjusted intensity for all post AtDCS measurements. This allowed us to compare potentiation effects of AtDCS at rest and after training. After applying stimulation for 7 minutes, we recorded 10 MEPs (at s1mV or adjusted s1mV intensities) every 5 minutes until 30 minutes post AtDCS application. At each time point, we averaged the recorded 10 MEP amplitudes and normalized these values to the average of 10 MEP amplitudes prior to AtDCS application.

To quantify the magnitude of occlusion, we calculated the occlusion index (OI) for each participant. We selected the peak MEP amplitude after AtDCS application normalized to the MEP amplitude prior to AtDCS application for each subject. The peak MEP amplitude was selected as the largest mean MEP on any one of the six post measurement time points. This measurement was done after each session: Baseline (no training), Day1 and Day2. The OI represents a comparison between the rest and post training measures, thus we subtracted baseline day amplitudes to both training session amplitudes in equation 2:


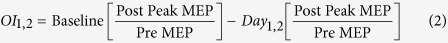


This measurement was used as an index of how much potentiation plasticity was used during training, where larger values for the OI are indicative of more occlusion, which would imply more resources were used to induce plasticity changes during learning.

#### Data Analysis

For all statistical data analysis, SPSS (IBM; Version 20) was used and effects were considered significant if *p* ≤ 0.05. All data are given as means ± SEM. We used separate polynomial nested repeated measures of ANOVA (ANOVA_RM_) for all behavioral and physiological measures. Furthermore, we used Mauchly’s sphericity test to validate that the variances between each group tested in ANOVA_RM_ are equal. When appropriate, Bonferroni corrected *post hoc* analysis was done to account for multiple comparisons.

We used skill measure as our primary behavioral outcome measure. To assess differences in early performance on Day 1, we used a one-way ANOVA_RM_ with between factor GROUP (Long, Short, Random) and within factor TIME (B1). Skill measure scores were also compared across participants who engaged in extended training (i.e. Long and Random). Here, we used ANOVA_RM_ with GROUP (Long, Random) as the between factor and DAY (Day1, Day2) and TIME (Block1, Block2…Block5) as within-factors.

To determine early changes in CBI, SICI, and s1MV, we used ANOVA_RM_ with GROUP (Long, Short, Random) as the between factor and DAY (Day1, Day2) and TIME (PRE, P1) as within factors. In a separate analysis, we assessed these TMS measurements when all stimulation time points were matched between GROUP (Long and Random) across DAY (Day1, Day2) and TIME (PRE, P1, P2, P3).

Using the peak-to-peak MEP amplitudes as the primary outcome measure, the amount of potentiation plasticity aftereffects via AtDCS application was compared using ANOVA_RM_ with the between factor GROUP (Long, Short, Random), and the within factors DAY (Base, Day1, Day2) and TIME (Pre-AtDCS, mean of Post-AtDCS [P1, P2, P3, P4, P5, P6]). Additionally, we used peak AtDCS response as our primary measure to assess how much LTP-like plasticity was used during training. For each day, peak MEP amplitudes aftereffects were compared using ANOVA_RM_ between each GROUP (Long, Short, Random) and within each DAY (Base, Day1, Day2).

To determine associations between early cerebellar physiological changes and behavior (Day1), we combined data from both Long and Short groups and performed a correlation analysis between early changes in CBI (P1-Pre) and early skill improvement within the first block (first 15 trials vs. next 15 trials). Similarly, to determine associations between OI_1_ and behavior (offline changes Day2 B1- Day1 B5) in the Long group, we also performed correlations using Spearman’s ρ.

## Additional Information

**How to cite this article:** Spampinato, D. and Celnik, P. Temporal dynamics of cerebellar and motor cortex physiological processes during motor skill learning. *Sci. Rep.* **7**, 40715; doi: 10.1038/srep40715 (2017).

**Publisher's note:** Springer Nature remains neutral with regard to jurisdictional claims in published maps and institutional affiliations.

## Supplementary Material

Supplementary Tables

## Figures and Tables

**Table 1:** M1 excitability measures.

	Day 1				Day 2			
	Pre	P1	P2	P3	Pre	P1	P2	P3
CBI (Unconditioned TS)								
Long	1.17 ± 0.08	1.10 ± 0.15	1.14 ± 0.12	1.20 ± 0.16	1.15 ± 0.09	1.17 ± 0.08	1.20 ± 0.12	1.13 ± 0.10
Short	1.11 ± 0.07	1.18 ± 0.14	—	—	1.13 ± 0.11	1.20 ± 0.13	—	—
Random	1.15 ± 0.11	1.17 ± 0.18	1.12 ± 0.12	1.17 ± 0.11	1.18 ± 0.09	1.21 ± 0.09	1.13 ± 0.13	1.23 ± 0.14
MEP								
Long	1.07 ± 0.09	1.39 ± 0.16	1.37 ± 0.15	1.46 ± 0.08	1.05 ± 0.11	1.34 ± 0.16	1.40 ± 0.17	1.37 ± 0.13
Short	1.06 ± 0.08	1.23 ± 0.19	—	—	1.03 ± 0.09	1.10 ± 0.12	—	—
Random	1.09 ± 0.12	1.26 ± 0.16	1.37 ± 0.21	1.38 ± 0.24	1.02 ± 0.13	1.21 ± 0.20	1.23 ± 0.25	1.39 ± 0.23
SICI Ratio								
Long	0.46 ± 0.07	0.45 ± 0.09	0.50 ± 0.10	0.65 ± 0.12	0.42 ± 0.08	0.51 ± 0.11	0.41 ± 0.12	0.47 ± 0.10
Short	0.41 ± 0.08	0.52 ± 0.10	—	—	0.45 ± 0.06	0.53 ± 0.09	—	—
Random	0.42 ± 0.16	0.40 ± 0.15	0.45 ± 0.14	0.41 ± 0.08	0.48 ± 0.13	0.41 ± 0.14	0.38 ± 0.13	0.41 ± 0.12

There were no differences in test MEP amplitudes during CBI assessment in any of the groups (adjusted stimulator output). When assessing corticomotor excitability using a fixed stimulation intensity (s1mV = intensity that elicited ~1 mV MEP at baseline), we found a significant increase in excitability over time for all groups, but no changes were associated with learning. Finally, we assessed short-latency intracortical inhibition to explore intrinsic inhibition within M1. We observed a reduction of SICI, in all groups, again indicating that changes were related to performance rather than learning. Data are means ± SEM.

## References

[b1] Dayan E. & Cohen L. G. Neuroplasticity subserving motor skill learning. Neuron 72, 443–454, doi: 10.1016/j.neuron.2011.10.008 (2011). 22078504 PMC3217208

[b2] Penhune V. B. & Steele C. J. Parallel contributions of cerebellar, striatal and M1 mechanisms to motor sequence learning. Behav Brain Res 226, 579–591, doi: 10.1016/j.bbr.2011.09.044 (2012). 22004979

[b3] Martin T. A., Keating J. G., Goodkin H. P., Bastian A. J. & Thach W. T. Throwing while looking through prisms. I. Focal olivocerebellar lesions impair adaptation. Brain 119(Pt 4), 1183–1198 (1996). 8813282 10.1093/brain/119.4.1183

[b4] Smith M. A. & Shadmehr R. Intact ability to learn internal models of arm dynamics in Huntington’s disease but not cerebellar degeneration. J Neurophysiol 93, 2809–2821, doi: 10.1152/jn.00943.2004 (2005). 15625094

[b5] Morton S. M. & Bastian A. J. Cerebellar contributions to locomotor adaptations during splitbelt treadmill walking. J Neurosci 26, 9107–9116, doi: 10.1523/jneurosci.2622-06.2006 (2006). 16957067 PMC6674518

[b6] Tseng Y. W., Diedrichsen J., Krakauer J. W., Shadmehr R. & Bastian A. J. Sensory prediction errors drive cerebellum-dependent adaptation of reaching. J Neurophysiol 98, 54–62, doi: 10.1152/jn.00266.2007 (2007). 17507504

[b7] Galea J. M., Vazquez A., Pasricha N., de Xivry J. J. & Celnik P. Dissociating the roles of the cerebellum and motor cortex during adaptive learning: the motor cortex retains what the cerebellum learns. Cereb Cortex 21, 1761–1770, doi: 10.1093/cercor/bhq246 (2011). 21139077 PMC3138512

[b8] Jayaram G., Galea J. M., Bastian A. J. & Celnik P. Human locomotor adaptive learning is proportional to depression of cerebellar excitability. Cereb Cortex 21, 1901–1909, doi: 10.1093/cercor/bhq263 (2011). 21239392 PMC3202738

[b9] Schlerf J. E., Galea J. M., Bastian A. J. & Celnik P. A. Dynamic modulation of cerebellar excitability for abrupt, but not gradual, visuomotor adaptation. J Neurosci 32, 11610–11617, doi: 10.1523/JNEUROSCI.1609-12.2012 (2012). 22915105 PMC3435878

[b10] Cantarero G. . Cerebellar direct current stimulation enhances on-line motor skill acquisition through an effect on accuracy. J Neurosci 35, 3285–3290, doi: 10.1523/JNEUROSCI.2885-14.2015 (2015). 25698763 PMC4331640

[b11] Floyer-Lea A. & Matthews P. M. Distinguishable brain activation networks for short- and long-term motor skill learning. J Neurophysiol 94, 512–518, doi: 10.1152/jn.00717.2004 (2005). 15716371

[b12] Lehericy S. . Distinct basal ganglia territories are engaged in early and advanced motor sequence learning. Proc Natl Acad Sci USA 102, 12566–12571, doi: 10.1073/pnas.0502762102 (2005). 16107540 PMC1194910

[b13] Seidler R. D. Differential effects of age on sequence learning and sensorimotor adaptation. Brain Res Bull 70, 337–346, doi: 10.1016/j.brainresbull.2006.06.008 (2006). 17027769

[b14] Steele C. J. & Penhune V. B. Specific increases within global decreases: a functional magnetic resonance imaging investigation of five days of motor sequence learning. J Neurosci 30, 8332–8341, doi: 10.1523/jneurosci.5569-09.2010 (2010). 20554884 PMC6634594

[b15] Imamizu H. . Human cerebellar activity reflecting an acquired internal model of a new tool. Nature 403, 192–195, doi: 10.1038/35003194 (2000). 10646603

[b16] Imamizu H., Kuroda T., Miyauchi S., Yoshioka T. & Kawato M. Modular organization of internal models of tools in the human cerebellum. Proc Natl Acad Sci USA 100, 5461–5466, doi: 10.1073/pnas.0835746100 (2003). 12704240 PMC154367

[b17] Graydon F. X., Friston K. J., Thomas C. G., Brooks V. B. & Menon R. S. Learning-related fMRI activation associated with a rotational visuo-motor transformation. Brain Res Cogn Brain Res 22, 373–383, doi: 10.1016/j.cogbrainres.2004.09.007 (2005). 15722208

[b18] Luaute J. . Dynamic changes in brain activity during prism adaptation. J Neurosci 29, 169–178, doi: 10.1523/jneurosci.3054-08.2009 (2009). 19129395 PMC6664918

[b19] Wessel M. J. . Enhancing Consolidation of a New Temporal Motor Skill by Cerebellar Noninvasive Stimulation. Cereb Cortex 26, 1660–1667, doi: 10.1093/cercor/bhu335 (2016). 25604611

[b20] Kim S., Ogawa K., Lv J., Schweighofer N. & Imamizu H. Neural Substrates Related to Motor Memory with Multiple Timescales in Sensorimotor Adaptation. PLoS Biol 13, e1002312, doi: 10.1371/journal.pbio.1002312 (2015). 26645916 PMC4672877

[b21] Karni A. . Functional MRI evidence for adult motor cortex plasticity during motor skill learning. Nature 377, 155–158, doi: 10.1038/377155a0 (1995). 7675082

[b22] Penhune V. B. & Doyon J. Cerebellum and M1 interaction during early learning of timed motor sequences. Neuroimage 26, 801–812, doi: 10.1016/j.neuroimage.2005.02.041 (2005). 15955490

[b23] Wu T., Kansaku K. & Hallett M. How self-initiated memorized movements become automatic: a functional MRI study. J Neurophysiol 91, 1690–1698, doi: 10.1152/jn.01052.2003 (2004). 14645385

[b24] Seidler R. D. . Neural correlates of encoding and expression in implicit sequence learning. Exp Brain Res 165, 114–124, doi: 10.1007/s00221-005-2284-z (2005). 15965762

[b25] Donchin O. . Cerebellar regions involved in adaptation to force field and visuomotor perturbation. J Neurophysiol 107, 134–147, doi: 10.1152/jn.00007.2011 (2012). 21975446

[b26] Muellbacher W., Ziemann U., Boroojerdi B., Cohen L. & Hallett M. Role of the human motor cortex in rapid motor learning. Exp Brain Res 136, 431–438 (2001). 11291723 10.1007/s002210000614

[b27] Reis J. . Noninvasive cortical stimulation enhances motor skill acquisition over multiple days through an effect on consolidation. Proc Natl Acad Sci USA 106, 1590–1595, doi: 10.1073/pnas.0805413106 (2009). 19164589 PMC2635787

[b28] Robertson E. M., Pascual-Leone A. & Miall R. C. Current concepts in procedural consolidation. Nat Rev Neurosci 5, 576–582, doi: 10.1038/nrn1426 (2004). 15208699

[b29] Richardson A. G. . Disruption of primary motor cortex before learning impairs memory of movement dynamics. J Neurosci 26, 12466–12470, doi: 10.1523/jneurosci.1139-06.2006 (2006). 17135408 PMC6674906

[b30] Medina J. F. & Lisberger S. G. Links from complex spikes to local plasticity and motor learning in the cerebellum of awake-behaving monkeys. Nat Neurosci 11, 1185–1192, doi: 10.1038/nn.2197 (2008). 18806784 PMC2577564

[b31] Yang Y. & Lisberger S. G. Role of plasticity at different sites across the time course of cerebellar motor learning. J Neurosci 34, 7077–7090, doi: 10.1523/jneurosci.0017-14.2014 (2014). 24849344 PMC4028490

[b32] Nudo R. J., Milliken G. W., Jenkins W. M. & Merzenich M. M. Use-dependent alterations of movement representations in primary motor cortex of adult squirrel monkeys. J Neurosci 16, 785–807 (1996). 8551360 10.1523/JNEUROSCI.16-02-00785.1996 PMC6578638

[b33] Kleim J. A. . Motor learning-dependent synaptogenesis is localized to functionally reorganized motor cortex. Neurobiol Learn Mem 77, 63–77, doi: 10.1006/nlme.2000.4004 (2002). 11749086

[b34] Kleim J. A., Barbay S. & Nudo R. J. Functional reorganization of the rat motor cortex following motor skill learning. J Neurophysiol 80, 3321–3325 (1998). 9862925 10.1152/jn.1998.80.6.3321

[b35] Rioult-Pedotti M. S., Donoghue J. P. & Dunaevsky A. Plasticity of the synaptic modification range. J Neurophysiol 98, 3688–3695, doi: 10.1152/jn.00164.2007 (2007). 17913995

[b36] Rioult-Pedotti M. S., Friedman D. & Donoghue J. P. Learning-induced LTP in neocortex. Science 290, 533–536 (2000). 11039938 10.1126/science.290.5491.533

[b37] Rioult-Pedotti M. S., Friedman D., Hess G. & Donoghue J. P. Strengthening of horizontal cortical connections following skill learning. Nat Neurosci 1, 230–234, doi: 10.1038/678 (1998). 10195148

[b38] Ziemann U., Ilic T. V., Pauli C., Meintzschel F. & Ruge D. Learning modifies subsequent induction of long-term potentiation-like and long-term depression-like plasticity in human motor cortex. J Neurosci 24, 1666–1672, doi: 10.1523/JNEUROSCI.5016-03.2004 (2004). 14973238 PMC6730462

[b39] Stefan K. . Temporary occlusion of associative motor cortical plasticity by prior dynamic motor training. Cereb Cortex 16, 376–385, doi: 10.1093/cercor/bhi116 (2006). 15930370

[b40] Rosenkranz K., Kacar A. & Rothwell J. C. Differential modulation of motor cortical plasticity and excitability in early and late phases of human motor learning. J Neurosci 27, 12058–12066, doi: 10.1523/JNEUROSCI.2663-07.2007 (2007). 17978047 PMC6673358

[b41] Lepage J. F. . Occlusion of LTP-like plasticity in human primary motor cortex by action observation. PLoS One 7, e38754, doi: 10.1371/journal.pone.0038754 (2012). 22701704 PMC3368919

[b42] Avanzino L. . Motor cortical plasticity induced by motor learning through mental practice. Front Behav Neurosci 9, 105, doi: 10.3389/fnbeh.2015.00105 (2015). 25972791 PMC4412065

[b43] Cantarero G., Lloyd A. & Celnik P. Reversal of long-term potentiation-like plasticity processes after motor learning disrupts skill retention. J Neurosci 33, 12862–12869, doi: 10.1523/JNEUROSCI.1399-13.2013 (2013). 23904621 PMC3728692

[b44] Cantarero G., Tang B., O’Malley R., Salas R. & Celnik P. Motor learning interference is proportional to occlusion of LTP-like plasticity. J Neurosci 33, 4634–4641, doi: 10.1523/JNEUROSCI.4706-12.2013 (2013). 23486938 PMC3727291

[b45] Anderson B. J., Alcantara A. A. & Greenough W. T. Motor-skill learning: changes in synaptic organization of the rat cerebellar cortex. Neurobiol Learn Mem 66, 221–229, doi: 10.1006/nlme.1996.0062 (1996). 8946414

[b46] Kleim J. A., Vij K., Ballard D. H. & Greenough W. T. Learning-dependent synaptic modifications in the cerebellar cortex of the adult rat persist for at least four weeks. J Neurosci 17, 717–721 (1997). 8987793 10.1523/JNEUROSCI.17-02-00717.1997 PMC6573226

[b47] Torriero S. . Changes in cerebello-motor connectivity during procedural learning by actual execution and observation. J Cogn Neurosci 23, 338–348, doi: 10.1162/jocn.2010.21471 (2011). 20350172

[b48] Pascual-Leone A. . Modulation of muscle responses evoked by transcranial magnetic stimulation during the acquisition of new fine motor skills. J Neurophysiol 74, 1037–1045 (1995). 7500130 10.1152/jn.1995.74.3.1037

[b49] Classen J., Liepert J., Wise S. P., Hallett M. & Cohen L. G. Rapid plasticity of human cortical movement representation induced by practice. J Neurophysiol 79, 1117–1123 (1998). 9463469 10.1152/jn.1998.79.2.1117

[b50] Wolpert D. M. & Kawato M. Multiple paired forward and inverse models for motor control. Neural Netw 11, 1317–1329 (1998). 12662752 10.1016/s0893-6080(98)00066-5

[b51] Diedrichsen J., White O., Newman D. & Lally N. Use-dependent and error-based learning of motor behaviors. J Neurosci 30, 5159–5166, doi: 10.1523/jneurosci.5406-09.2010 (2010). 20392938 PMC6632748

[b52] Baraduc P., Lang N., Rothwell J. C. & Wolpert D. M. Consolidation of dynamic motor learning is not disrupted by rTMS of primary motor cortex. Curr Biol 14, 252–256, doi: 10.1016/j.cub.2004.01.033 (2004). 14761660

[b53] Hadipour-Niktarash A., Lee C. K., Desmond J. E. & Shadmehr R. Impairment of retention but not acquisition of a visuomotor skill through time-dependent disruption of primary motor cortex. J Neurosci 27, 13413–13419, doi: 10.1523/jneurosci.2570-07.2007 (2007). 18057199 PMC6673085

[b54] Schlerf J. E., Galea J. M., Spampinato D. & Celnik P. A. Laterality Differences in Cerebellar-Motor Cortex Connectivity. Cereb Cortex 25, 1827–1834, doi: 10.1093/cercor/bht422 (2015). 24436320 PMC4459286

[b55] Jayaram G. . Modulating locomotor adaptation with cerebellar stimulation. J Neurophysiol 107, 2950–2957, doi: 10.1152/jn.00645.2011 (2012). 22378177 PMC3378372

[b56] Herzfeld D. J. . Contributions of the cerebellum and the motor cortex to acquisition and retention of motor memories. Neuroimage 98, 147–158, doi: 10.1016/j.neuroimage.2014.04.076 (2014). 24816533 PMC4099269

[b57] Alonzo A., Brassil J., Taylor J. L., Martin D. & Loo C. K. Daily transcranial direct current stimulation (tDCS) leads to greater increases in cortical excitability than second daily transcranial direct current stimulation. Brain stimulation 5, 208–213, doi: 10.1016/j.brs.2011.04.006 (2012). 22037139

[b58] Galvez V., Alonzo A., Martin D. & Loo C. K. Transcranial direct current stimulation treatment protocols: should stimulus intensity be constant or incremental over multiple sessions? The international journal of neuropsychopharmacology 16, 13–21, doi: 10.1017/s1461145712000041 (2013). 22310245

[b59] Jacobs K. M. & Donoghue J. P. Reshaping the cortical motor map by unmasking latent intracortical connections. Science 251, 944–947 (1991). 2000496 10.1126/science.2000496

[b60] Malinow R., Mainen Z. F. & Hayashi Y. LTP mechanisms: from silence to four-lane traffic. Curr Opin Neurobiol 10, 352–357 (2000). 10851179 10.1016/s0959-4388(00)00099-4

[b61] Butefisch C. M. . Mechanisms of use-dependent plasticity in the human motor cortex. Proc Natl Acad Sci USA 97, 3661–3665, doi: 10.1073/pnas.050350297 (2000). 10716702 PMC16296

[b62] Perez M. A., Lungholt B. K., Nyborg K. & Nielsen J. B. Motor skill training induces changes in the excitability of the leg cortical area in healthy humans. Exp Brain Res 159, 197–205, doi: 10.1007/s00221-004-1947-5 (2004). 15549279

[b63] Coxon J. P., Peat N. M. & Byblow W. D. Primary motor cortex disinhibition during motor skill learning. J Neurophysiol 112, 156–164, doi: 10.1152/jn.00893.2013 (2014). 24717346

[b64] Huang V. S., Haith A., Mazzoni P. & Krakauer J. W. Rethinking motor learning and savings in adaptation paradigms: model-free memory for successful actions combines with internal models. Neuron 70, 787–801, doi: 10.1016/j.neuron.2011.04.012 (2011). 21609832 PMC3134523

[b65] Haith A. M. & Krakauer J. W. Model-based and model-free mechanisms of human motor learning. Adv Exp Med Biol 782, 1–21, doi: 10.1007/978-1-4614-5465-6_1 (2013). 23296478 PMC3570165

[b66] Wymbs N. F., Bastian A. J. & Celnik P. A. Motor Skills Are Strengthened through Reconsolidation. Current biology: CB 26, 338–343, doi: 10.1016/j.cub.2015.11.066 (2016). 26832444 PMC4747782

[b67] Rossini P. M. . Non-invasive electrical and magnetic stimulation of the brain, spinal cord, roots and peripheral nerves: Basic principles and procedures for routine clinical and research application. An updated report from an I.F.C.N. Committee. Clin Neurophysiol 126, 1071–1107, doi: 10.1016/j.clinph.2015.02.001 (2015). 25797650 PMC6350257

[b68] Liebetanz D., Nitsche M. A., Tergau F. & Paulus W. Pharmacological approach to the mechanisms of transcranial DC-stimulation-induced after-effects of human motor cortex excitability. Brain 125, 2238–2247 (2002). 12244081 10.1093/brain/awf238

[b69] Nitsche M. A. . Facilitation of implicit motor learning by weak transcranial direct current stimulation of the primary motor cortex in the human. J Cogn Neurosci 15, 619–626, doi: 10.1162/089892903321662994 (2003). 12803972

[b70] Fritsch B. . Direct current stimulation promotes BDNF-dependent synaptic plasticity: potential implications for motor learning. Neuron 66, 198–204, doi: 10.1016/j.neuron.2010.03.035 (2010). 20434997 PMC2864780

[b71] Dyke K., Kim S., Jackson G. M. & Jackson S. R. Intra-Subject Consistency and Reliability of Response Following 2 mA Transcranial Direct Current Stimulation. Brain stimulation, doi: 10.1016/j.brs.2016.06.052 (2016). 27387569

[b72] Horvath J. C., Vogrin S. J., Carter O., Cook M. J. & Forte J. D. Effects of a common transcranial direct current stimulation (tDCS) protocol on motor evoked potentials found to be highly variable within individuals over 9 testing sessions. Experimental brain research 234, 2629–2642, doi: 10.1007/s00221-016-4667-8 (2016). 27150317

[b73] Lopez-Alonso V., Cheeran B., Rio-Rodriguez D. & Fernandez-Del-Olmo M. Inter-individual variability in response to non-invasive brain stimulation paradigms. Brain stimulation 7, 372–380, doi: 10.1016/j.brs.2014.02.004 (2014). 24630849

[b74] Lopez-Alonso V., Fernandez-Del-Olmo M., Costantini A., Gonzalez-Henriquez J. J. & Cheeran B. Intra-individual variability in the response to anodal transcranial direct current stimulation. Clinical neurophysiology: official journal of the International Federation of Clinical Neurophysiology 126, 2342–2347, doi: 10.1016/j.clinph.2015.03.022 (2015). 25922127

[b75] Ugawa Y., Uesaka Y., Terao Y., Hanajima R. & Kanazawa I. Magnetic stimulation over the cerebellum in humans. Ann Neurol 37, 703–713, doi: 10.1002/ana.410370603 (1995). 7778843

[b76] Galea J. M., Jayaram G., Ajagbe L. & Celnik P. Modulation of cerebellar excitability by polarity-specific noninvasive direct current stimulation. J Neurosci 29, 9115–9122, doi: 10.1523/JNEUROSCI.2184-09.2009 (2009). 19605648 PMC2760225

[b77] Pinto A. D. & Chen R. Suppression of the motor cortex by magnetic stimulation of the cerebellum. Exp Brain Res 140, 505–510, doi: 10.1007/s002210100862 (2001). 11685404

[b78] Daskalakis Z. J. . Exploring the connectivity between the cerebellum and motor cortex in humans. J Physiol 557, 689–700, doi: 10.1113/jphysiol.2003.059808 (2004). 15047772 PMC1665103

[b79] Kujirai T. . Corticocortical inhibition in human motor cortex. J Physiol 471, 501–519 (1993). 8120818 10.1113/jphysiol.1993.sp019912 PMC1143973

[b80] Ziemann U., Rothwell J. C. & Ridding M. C. Interaction between intracortical inhibition and facilitation in human motor cortex. J Physiol 496(Pt 3), 873–881 (1996). 8930851 10.1113/jphysiol.1996.sp021734 PMC1160871

[b81] Heise K. F. . Differential behavioral and physiological effects of anodal transcranial direct current stimulation in healthy adults of younger and older age. Front Aging Neurosci 6, 146, doi: 10.3389/fnagi.2014.00146 (2014). 25071555 PMC4091308

