# Supplementary material for: Temporal dynamics of cerebellar and motor cortex physiological processes during motor skill learning
Source: Sci Rep. 2017 Jan 16;7:40715. doi: 10.1038/srep40715 (PMC5238434; doi:10.1038/srep40715)
Supplement: Supplementary Tables [file srep40715-s1.pdf]

Temporal dynamics of cerebellar and motor cortex physiological processes during motor skill learning

D. Spampinato[1,2] and P. Celnik[2,3,4]*
[1]Department of Biomedical Engineering, [2]Department of Physical Medicine and Rehabilitation, [3]Department of Neuroscience, and [4]Department of Neurology, Johns Hopkins School of Medicine

Supplementary Tables

Supplementary Table S1. rMT and TS intensity

	Day 1	Day 2	Paired t-test	
rMT			T	P-value
Long	40.9 ± 1.7	40.7 ± 1.5	t(9) = 0.56	*p* = 0.59
Short	41.8 ± 1.7	41.9 ± 1.8	t(10) = 0.23	*p* = 0.82
Random	40.5 ± 1.9	40.3 ± 2.2	t(7) = 0.61	*p* = 0.56
Unadjusted TS			T	P-value
Long	49.4 ± 1.6	49.0 ± 1.7	t(9) = 0.35	*p* = 0.73
Short	51.8 ± 2.1	52.1 ± 2.3	t(10) = 0.31	*p* = 0.76
Random	49.4 ± 2.5	49.2 ± 2.8	t(7) = 0.25	*p* = 0.81

Supplementary Table S2. Adjusted TS intensity for CBI and SICI

	Day 1				Day 2			
	Pre	P1	P2	P3	Pre	P1	P2	P3
Adjusted TS MSO%								
Long	-	48.9 ± 2.1	48.7 ± 1.9	49.6 ± 1.9	-	48.5 ± 1.6	48.5 ± 1.6	48.4 ± 1.7
Short	-	51.6 ± 2.0	-	-	-	51.3 ± 1.9	-	-
Random	-	48.6 ± 2.7	48.8 ± 2.5	48.9 ± 2.5	-	49.0 ± 2.8	48.6 ± 2.9	48.8 ± 2.8
